# Evaluation of the hypoglycemic and hypolipidemic effects of an ethylacetate fraction of *Artocarpus heterophyllus* (jak) leaves in streptozotocin-induced diabetic rats

**DOI:** 10.4103/0973-1296.66933

**Published:** 2010

**Authors:** S. Chackrewarthy, M. I. Thabrew, M. K. B. Weerasuriya, S. Jayasekera

**Affiliations:** Department of Biochemistry and Clinical Chemistry, Faculty of Medicine, University of Kelaniya, PO Box 6, Thalagolla Road, Ragama, Sri Lanka; 1 Institute of Biochemistry, Molecular Biology and Biotechnology, University of Colombo, Colombo 3, Sri Lanka; 2 Department of Chemistry, Faculty of Science, University of Kelaniya, Dalugama, Kelaniya, Sri Lanka; 3 Animal Center, Medical Research Institute, Colombo 8, Sri Lanka

**Keywords:** *Artocarpus heterophyllus*, ethylacetate, hypoglycemic activity, hypolipidemic, streptozotocin

## Abstract

Aqueous extracts of mature leaves of *Artocarpus heterophyllus* (jak) are used by traditional medical practitioners in Sri Lanka and India for the treatment of diabetes. This study was conducted to investigate the hypoglycemic and hypolipidemic effects of an ethylacetate (EA) fraction of the mature leaves of *A. heterophyllus* in a streptozotocin (STZ) induced diabetic rat model. In normoglycemic rats, administration of a single dose (20 mg/kg) of the EA fraction resulted in a significant (*P* < 0.05) reduction in the fasting blood glucose concentration and a significant improvement in glucose tolerance (*P* < 0.05), compared to the controls. In STZ-induced diabetic rats, chronic administration of the EA fraction of *A. heterophyllus* leaves daily for 5 weeks resulted in a significant lowering of serum glucose, cholesterol and triglyceride (TG) levels. Compared to control diabetic rats, the extract-treated rats had 39% less serum glucose, 23% lower serum total cholesterol and 40% lower serum TG levels and 11% higher body weight at the end of the fifth week. The percentage reductions in the serum parameters mediated by the test fraction were comparable with those produced by glibenclamide (0.6 mg/kg), the reference drug used in this study. It can be concluded that the EA fraction of *A. heterophyllus* leaves contains one or more hypoglycemic and hypolipidemic principles which have the potential to be developed further for the treatment of diabetes specifically associated with a hyperlipidemic state.

## INTRODUCTION

*Artocarpus heterophyllus* (family Moraceae), commonly known as jak, is a tropical plant native to south and southeast Asia. In Sri Lanka and India, aqueous extracts of mature jak leaves are used by traditional medical practitioners as a treatment for diabetes mellitus.[1,2] Diabetes mellitus is a syndrome characterized by chronic hyperglycemia and disturbances of fat and protein metabolism associated with absolute or relative deficiencies in insulin secretion and/or insulin action.[3]

Previous investigations have shown that aqueous extracts of *A. heterophyllus* leaves possess significant hypoglycemic activity,[4] and improve the glucose tolerance in normal human subjects and in maturity onset diabetic patients.[5] Recently, it has been reported that the hypoglycemic activity of *A. heterophyllus* leaves is due to a flavonoid fraction isolated from hot water extract of the leaves.[6] In a preliminary investigation with normoglycemic rats carried out by the authors (unpublished data) to test the hypoglycemic potential of fractions separated from *A. heterophyllus* leaves by sequential fractionation, the ethylacetate (EA) fraction and the aqueous fraction were found to exert the highest hypoglycemic effects. However, of the two fractions, EA fraction exerted a significantly (*P* < 0.05) greater hypoglycemic effect than the aqueous fraction. While the hypoglycemic properties of *A. heterophyllus* leaves are well reported, there are no reports on the effects of the leaf extract on hyperlipidemia associated with diabetes despite the clinical relevance of such studies.

There is growing interest in plants with both hypoglycemic and hypolipidemic properties since they have the potential to be developed further for treatment of diabetes specially associated with a hyperlipidemic state (St. Louis, MO USA). Many plants with hypoglycemic properties, which can also exert hypolipidemic effects have been reported in the literature.[7,8] Therefore, in the present study, the effects of the EA fraction of *A. heterophyllus* leaves on (a) glucose tolerance in normoglycemic rats and (b) glucose and lipid levels of streptozotocin (STZ) induced diabetic rats, have been investigated with the aim of understanding the hypoglycemic and hypolipidemic potential of these leaves.

## MATERIALS AND METHODS

### Chemicals

All analytical grade reagents for the fractionation were purchased from Hemsons International (Pte) Ltd. (Colombo, Sri Lanka). STZ was purchased from Sigma Chemical Company St. Louis, MOUSA). Glibenclamide was purchased from the State Pharmaceutical Company (Colombo, Sri Lanka).

### Experimental animals

Male Wistar rats of average body weight (BW) 200 ± 20 g, obtained from the Medical Research Institute, Colombo, were maintained under standard conditions and used for the study.

### Plant material

Mature leaves of *A. heterophyllus* were collected from a tree growing in Kelaniya, Sri Lanka. They were identified by a Botanist at the Bandaranayake Memorial Ayurveda Institute, Nawinna, Sri Lanka. A voucher specimen of the *A. heterophyllus* leaves has been deposited at the Department of Biochemistry (Kln/Biochem/01).

### Fractionation of *A. hetrophyllus* leaf extract

Two hundred grams of fresh mature leaves of *A. hetrophyllus* were cut into small pieces and sequentially extracted using three different solvents with increasing polarity. The water insoluble components were first extracted by boiling the leaves with 1 l of dichloromethane for 3 h in a soxhlet extractor. The residue remaining after dichloromethane extraction was then subject to extraction with EA using the same procedure. The EA insoluble material was finally subject to water extraction in the same manner. The supernatants obtained from the above extractions were concentrated and evaporated to dryness. The EA extract (1.1% w/w) was used for this investigation.

### Effect of ethylacetate fraction on glucose tolerance

Rats were randomly assigned to four groups of six rats in each and were fasted overnight. Blood samples were drawn from tail veins for the determination of fasting blood glucose levels. Then, group I(control) was orally administered distilled water (10 ml/kg), while groups II and III were administered the EA extract (10 and 20 mg/kg, respectively). Group IV was orally administered the reference drug, glibenclamide (0.6 mg/kg). After 30 minutes, an oral dose of glucose (50% w/v, 10 ml/kg) was given and blood samples were collected at 1 h intervals for 3 h.

### Induction of diabetes in rats

Diabetes was induced in rats by single intravenous injection of freshly prepared STZ solution (in 0.1 M citrate buffer pH = 4.5) at a dose of 60 mg/kg bw. Diabetes was developed and stabilized in STZ-treated rats over a period of 3 days. Rats with a fasting plasma glucose level of 200 mg/dl or above were included in the study.

### Evaluation of the effects of the ethylacetate fraction on blood glucose and lipid levels of streptozotocin-induced diabetic rats

Diabetic rats were divided into three groups of six rats in each (control, test and reference groups). All the rats were fasted overnight and blood samples were drawn from the tail vein for the determination of baseline data. Diabetic control and the normal control groups were orally administered distilled water (10 ml/kg), while the diabetic test group and the diabetic reference group were orally administered the EA fraction (20 mg/kg) and glibenclamide (0.6 mg/kg), respectively, daily for 5 weeks. After periods of 2, 4 and 5 weeks, blood was drawn from the tail vein of fasting rats and assayed for glucose, cholesterol and triglycerides (TGs) using commercially available reagent kits purchased from Randox Laboratories Ltd. Crumlin, Co Antrim (UK).

### Statistical analysis

The data were expressed as mean ± SEM. Data were analyzed using STATA (version 8.2). A level of P < 0.05 was considered to be significant.

## RESULTS

### Effect of the ethylacetate fraction on glucose tolerance of normoglycemic rats

According to results of preliminary experiments carried out by the authors (unpublished results) with normoglycemic rats, both EA and aqueous fractions of *Artocarpus* leaves can significantly lower fasting blood glucose levels by 42.5 and 28.7%, respectively, at 2 h post administration of a single dose (50 mg/kg) of each fraction. In glucose tolerance studies carried out in the present investigation, rats receiving the EA fraction showed a significant improvement in their ability to utilize the external glucose load compared to the control group [Table 1]. The peak increase in blood glucose concentration was observed at 2 h post glucose administration.

**Table 1:** Effect of EA fraction on glucose tolerance of normoglycemic rats

Groups	Serum glucose concentration (mg/dl)		% Reduction in serum glucose compared to control
	Fasting	2 h post glucose	
Control	95.1 ± 4.4	169.5 ± 3.6	
EA fraction (10 mg/kg)	96.5 ± 2.3	137.3 ± 2.7	18.9*
EA fraction (20 mg/kg)	98.6 ± 3.5	124.8 ± 4.2	26.4*
Glibenclamide (0.6 mg/kg)	98.3 ± 1.9	108.5 ± 2.9	35.9*

Means ± SD

* *P* < 0.05 compared to control

**Table 2:** Serum glucose levels in STZ-induced diabetic rats after prolonged treatment with EA fraction

Groups	Post STZ	2 weeks	4 weeks	5 weeks
Normal control	98.4 ± 2.9	90.6 ± 5.7	91.6 ± 4.8	92.7 ± 6.6
Diabetic control	254.3 ± 2.5	245.5 ± 6.6^a^	250.0 ± 3.9^a^	253.4 ± 6.3 a
Diabetic + EA fraction (20 mg/kg bw)	252.8 ± 11.9	189.4 ± 5.1^b^	160.1 ± 11.0^b^	154.2 ± 11.1^b^
Diabetic+ glibenclamide (0.6 mg/kg bw)	235.2 ± 8.3	166.4 ± 4.8	126.6 ± 9.2	112.3 ± 7.5

Mean serum glucose levels in mg/dl ± SD, Significantly different (*P* < 0.05)

a compared to normal controls

b compared to diabetic controls

### Effect of ethylacetate fraction on the fasting blood glucose levels of streptozotocin-induced diabetic rats

Chronic administration [Table 2] of the EA fraction caused a significant fall (*P* < 0.05) in the fasting blood sugar levels of diabetic rats when compared with diabetic controls which did not receive the EA fraction. This is evident in the second week itself and the reduction in the fasting blood sugar in the EA fraction treated rats (22.8%) was fairly comparable to that produced by the reference drug glibenclamide (32.2%) during the same time period. As in the glibenclamide-treated rats, in rats receiving the EA fraction also, the fall in the blood sugar level continued progressively till the end of the fifth week.

### Effect of the ethylacetate fraction on serum lipid levels and body weights of streptozotocin-induced diabetic rats

Alterations in the lipid profiles and BWs of STZ-induced diabetic rats are summarized in Table 3. The increased serum cholesterol and TGs in diabetic rats when compared with normal control rats is an evidence for development of hyperlipidemia in diabetic rats. Treatment of diabetic rats with the EA fraction resulted in a significant fall (*P* < 0.05) in the levels of both total cholesterol (TC) and TGs compared to diabetic controls. The fall in serum TG levels (40%) was more marked than that of cholesterol levels (23%). As evident from Table 3, the EA fraction had an improving effect on the BW (11%) of diabetic rats, which was restored to near normal levels.

## DISCUSSION

In a preliminary study carried out by the authors, the greater hypoglycemic activity mediated by the EA fraction when compared with that produced by the aqueous fraction (42.5 and 28.7% reduction of blood glucose levels, respectively, at 2 h post treatment) is possibly due to higher solubility of the active hypoglycemic compounds in EA. This result supports the previous findings of Chandrika *et al*.[6] that hypoglycemic activity of *A. heterophyllus* leaf extract is mediated mainly by flavonoids, which have a higher solubility in EA. Hence, in the present study, the effects of an EA fraction of *A. heterophyllus* leaves on (a) the glucose tolerance of normoglycemic rats and (b) blood glucose and lipid levels of STZ-induced diabetic rats have been investigated. Results of the glucose tolerance test provide confirmatory evidence for the ability of the EA fraction to mediate hypoglycemic effects in normoglycemic rats. In EA fraction treated rats, there was a significant improvement in their ability to utilize the external glucose load in a dose-dependant manner and a dosage of 20 mg/kg was found to be more effective than the dose of 10 mg/kg [Table 1].

Findings of the present study also revealed that the EA fraction can mediate significant reductions in blood glucose and lipid levels in STZ-induced diabetic, hyperlipidemic rats. According to Aybar *et al*.[9] a low dose of STZ (60 mg/kg) produces an incomplete destruction of pancreatic β-cells, even though the rats became permanently diabetic. In EA fraction treated diabetic rats, serum glucose levels progressively decreased till the end of the fifth week [Table 2]. Glibenclamide stimulates insulin secretion from pancreatic β-cells.[10] Therefore, it may be suggested that stimulation of insulin release from the still functioning β-cells by active principles in EA fraction may be one of the mechanisms by which this fraction mediates its hypoglycemic effect, as proposed for some other plant extracts.[11,12] Further, components of the EA fraction as proposed by Gomes *et al*.[13] with reference to *Camellia sinensis* (black tea), may be able to generate β-cells of the pancreas or protect the intact β-cells from further deterioration so that they may remain active and continue to produce insulin. The above effects with respect to the EA fraction require further investigation.

Hyperlipidemia is a metabolic complication of both clinical and experimental diabetes.[14] The levels of serum TC and TGs were significantly decreased in diabetic rats treated with the EA fraction compared to diabetic controls (*P* < 0.05) [Table 3]. However, there was a significantly (*P* < 0.01) greater decrease in the TG level than that of TC level. This effect on diabetic hypertriglyceridemia in EA fraction treated rats could be due to improved glycemic control. The improved glycemic control by sulfonylureas accompanied by decreased serum very low density lipoprotein (VLDL) and TG levels has already been reported.[15] The reduction in cholesterol levels in test rats could be due to inhibitory effect of the active principles on enzymes of cholesterol biosynthesis[16,17] and/or due to the enhanced uptake of low density lipoprotein (LDL) cholesterol by extrahepatic tissues. To obtain confirmatory evidence of these views, further studies on the effects of the EA fraction on LDL and high density lipoprotein (HDL) cholesterol levels need to be conducted. A high fat intake and increased levels of free fatty acids in circulation have been implicated in the development of insulin resistance.[18,19] Based on these observations, it could be speculated that the EA fraction mediated reduction in circulating levels of TGs may also help to ameliorate insulin resistance in diabetic rats and thereby stimulate glucose utilization by peripheral tissues.[20]

**Table 3:** Serum TC, TG levels and BW in STZinduced diabetic rats after 5 weeks of treatment with EA fraction

Groups	TC (mg/dl)	TG (mg/dl)	BW (g)
Normal control	78.2 ± 3.8	102.2 ± 2.3	201.0 ± 8.5
Diabetic control	127.9 ± 3.2^a^	199.8 ± 6.2^a^	172.1 ± 5.5
Diabetic + EA fraction (20 mg/kg bw)	98.5 ± 4.3^b^	119.0 ± 2.5^b^	190.4 ± 1.2
Diabetic + glibenclamide (0.6 mg/kg bw)	87.4 ± 3.2	116.8 ± 1.3	196.5 ± 3.3

Means ± SD, Significantly different (*P* < 0.05)

a compared to normal controls

b compared to diabetic controls

The weight loss observed in diabetic rats, restored to near normal levels by treatment with EA fraction, may be a reflection of improved health resulting from the effects of the EA fraction on insulin release.

In conclusion, the data obtained from the present study indicate that EA fraction of *A. heterophyllus* leaves may contain one or more active principles which produce significant hypoglycemic effect, lowers both TC and TGs and improves the weight loss in STZ-induced diabetic rats. This investigation reveals that the EA fraction of *A. heterophyllus* leaves has the potential to be developed further into a natural anti-diabetic drug that can exert both hypoglycemic and hypolipidemic effects.
